# Comparison of sampling and culture methods for the recovery of yeast from hospital surfaces

**DOI:** 10.1017/ash.2024.481

**Published:** 2025-01-17

**Authors:** Allison R. Eberly, Alyssa M. Valencia, Kate Peacock, David McDonald, Tiffany Hink, Carleigh Samuels, Lucy Vogt, Yao-Peng Xue, Maggie Newberry, Caroline A. O’Neil, Gautam Dantas, Jennie H. Kwon

**Affiliations:** 1 Division of Laboratory and Genomic Medicine, Department of Pathology and Immunology, Washington University in St. Louis, St. Louis, MO, USA; 2 Division of Infectious Diseases, Department of Internal Medicine, Washington University in St. Louis, St. Louis, MO, USA; 3 The Edison Family Center for Genome Sciences and Systems Biology, Washington University School of Medicine in St Louis, St Louis, MO, USA; 4 Department of Biomedical Engineering, Washington University in St Louis, St Louis, MO, USA; 5 Department of Molecular Microbiology, Washington University School of Medicine in St Louis, St Louis, MO, USA; 6 Department of Pediatrics, Washington University School of Medicine in St Louis, St Louis, MO, USA

## Abstract

**Objective:**

To compare the recovery of yeast from hospital surfaces from two different collection methods: Eswab moistened with molecular water, and premoistened stick-mounted sponge.

**Design:**

Comparison of collection methods for the recovery of yeast in the hospital environment.

**Setting:**

This study took place at intensive care units of a large academic medical center.

## Introduction

Fungal infections caused by drug-resistant *Candida* species can cause life-threatening infections in hospitalized patients.^1^ Although rates of healthcare-associated invasive fungal infections (HA-IFIs) continue to increase, there is limited data about methods to detect and reduce the burden of fungal organisms in the healthcare environment.^2^ Furthermore, there is growing evidence that clinically significant fungi can persist in the environment, acting as a reservoir for potential transmission to vulnerable patients.^3,4^ To address this gap, the objective of this project was to compare and optimize sampling and culture methods for yeast recovery from hospital surfaces.

## Sample collection

This study took place at Barnes-Jewish Hospital (BJH) in St. Louis, MO. This study was approved by the Washington University Human Research Protection Office (#202209127). High-touch surfaces were sampled at a single time point using Eswabs (Copan, Brescia, Italy) and sponge sticks (3M, St. Paul, MN) in 10 patient rooms in the medical intensive care unit (MICU) and 10 patient rooms in the surgical ICU (SICU). Stick-mounted sponges come premoistened in bags for sampling and firm pressure was applied to surfaces being sampled. Eswabs were premoistened by dipping them into molecular-grade sterile water to mimic that of the stick-mounted sponge. Surfaces were swabbed by applying firm pressure to the area being sampled and then placed back into individual vials of transport media as previously described.^5^

In each patient room, 7 high-touch areas within individual patient rooms were chosen to conduct sampling: 1) sink and sink drain composite, 2) floor composite, 3) bathroom composite, 4) ventilator (if present) and IV pole composite, 5) visitor chair and/or bed composite, 6) ≤3 feet from the patient (bedside rail, call button, phone, and table), and 7) >3 feet from the patient (inner door handle, keyboard, and barcode scanner). Eight additional high-touch area samples from communal spaces were collected in both ICU settings: 1) visitor bathroom composite, 2) visitor sink composite, 3) floor composite, 4) chairs, table, and vending machine composite, 5) glucometer, 6) Doppler ultrasound, 7) vital signs monitor, and 8) medication dispenser. Sample sites were chosen based on previously published studies about bacterial load in specific areas of hospital rooms.^6^ Each site was sampled by both the Eswab and stick-mounted sponge by the same individual. For example, the floor composite sample for the Eswab consisted of a 4-inch by 4-inch area near the patient waist area along the bed and then a second 4-inch by 4-inch area next to the IV pole. The floor composite collection was repeated for the sponge-mounted stick, using the same parameters, near but not overlapping the Eswab collection areas.

## Sample processing and culture methods

Samples using stick-mounted sponges were processed as previously published in Rose *et al*.,^7^ with the following modifications: stick-mounted sponges were placed into a stomacher bag with 10 mL of phosphate-buffered saline containing 0.02% Tween 80 (PBST) and processed with a stomacher (Biomaster, Seward, Bohemia, NY) for 1 minute at 265 rpm. After processing via the stomacher and settling for 5–10 minutes, specimens were transferred into 15 mL conical tubes. The samples were centrifuged at 3000 g for 15 minutes at room temperature to pellet cellular material. Supernatant was removed after centrifugation and pellets resuspended in the remaining 3 mL volume for culture.

Samples collected using Eswabs were processed by vortex mixing for 30 seconds prior to plating the specimens for culture.

All samples were cultured using 100 uL of eluate cross-streaked onto one tryptic soy agar with 5% sheep blood (BAP, Hardy Diagnostics, Santa Maria, CA) plate and two Sabouraud dextrose with chloramphenicol agar (SABC, Hardy Diagnostics, Santa Maria, CA) plates. BAP agar and one SABC agar were incubated at 35^o^C and one SABC incubated at 30^o^C. BAP and SABC plates were screened for yeast colonies at days 1 and 2 via colony morphology and wet mount light microscopy of colonies. The SABC was incubated for a total of 7 days and screened on days 1, 2, and 7 for yeast colonies. Yeast isolates were subcultured to SABC and incubated at 30^o^C for isolation and identified using matrix-assisted laser desorption/ionization-time of flight mass spectrometry (MALDI-TOF) performed on the Vitek MS with database V3.2 (bioMerieux, Marcy-l’ Étoile, France).

## Results

**Figure 1.**
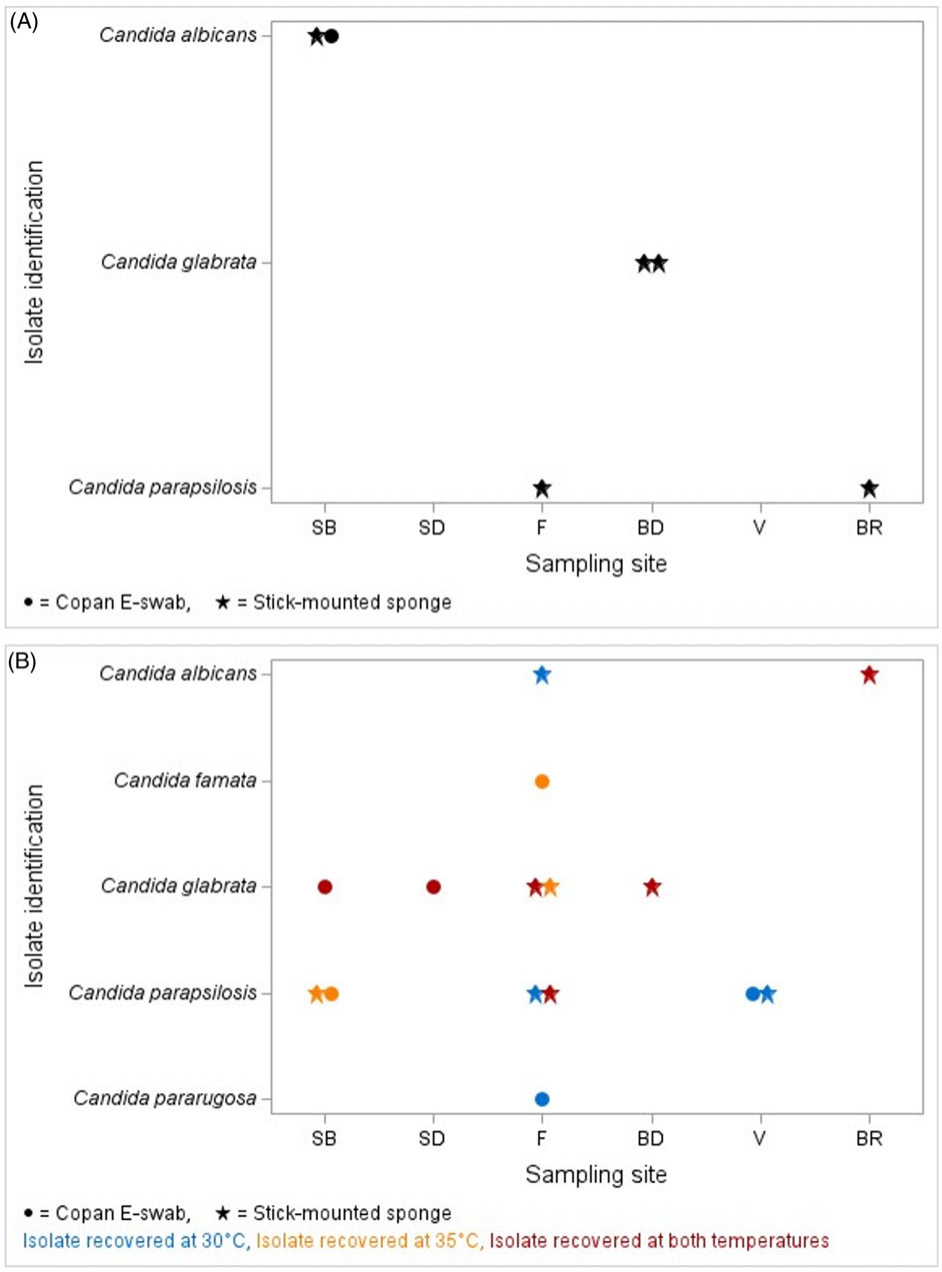
A. *Candida* species isolated from the sampling of 10 high-touch surfaces. SABC and BAP plates were incubated at 35^o^C. Locations from which yeast were not recovered are not shown on the graph. B. Fifteen *Candida* species isolates recovered from SABC and BAP plates were incubated at 35^o^C with the addition of SABC plate incubated at 30^o^C. Locations from which yeast were not recovered are not shown on the graph. Each data point represents a unique sampling site. Abbreviations: SB, sink bowl; SD, sink drain; F floor composite; BD, bathroom door handle, toilet handle, toilet seat; V, visitor chair or bed; BR, bedside rail, call button, phone, table.

During the method evaluation period, 633 samples were collected (299 from sponge stick, 334 from Eswab). Of these, 3.3% (21/633) were positive for the growth of *Candida* species. Figure 1A shows the 6 total *Candida* species recovered during the first sampling of 10 high-touch surfaces of the MICU and SICU for which plates were incubated at 35^o^C only. Only 1 of the 5 positive locations (sink bowl, SB) had the same yeast recovered by both the Eswab and stick-mounted sponge; the remaining 4 positive locations had yeast recovered by stick-mounted sponge only. Figure 1B shows the 15 *Candida* species isolates recovered during the second sampling with the addition of SABC plate incubated at 30^o^C. The 15 data points represent 15 unique *Candida* species isolated from a single sampling site. For example, the 7 *Candida* isolates recovered from the floor were from 7 different patient rooms out of the 10 total patient rooms sampled. The surfaces with the highest recovery of yeast from both samplings were the floor composite samples (1.3%, 8/633) and the bathroom door handle, toilet handle, and toilet seat samples (0.5%, 3/633). Sponge sticks had a higher yield of *Candida* species isolation with 14 isolates recovered versus Eswabs that recovered 7 isolates. The addition of the second SABC plate incubated at 30^o^C increased the isolate recovery with the addition of 6 more isolates recovered. *Candida parapsilosis* was the most common species isolated.

## Discussion

Fungal infections continue to increase in prevalence within the hospital setting.^1^ For example, the number of infections caused by *Candida auris*, including isolates resistant to all three classes of antifungals, are continuing to rise. Therefore, the ability to be able to have standardized surveillance and investigation studies for the recovery of *Candida* species, especially *C. auris*, is important for infection prevention. There are studies that report the use of sponge sticks for the recovery of yeast.^2,8,9^ However, for ease of use and collection, in part due to the larger size of the stick-mounted sponges, we wanted compare the recovery of stick-mounted sponges to Eswabs, which are readily stocked in hospital settings. We report a single time point of hospital environmental sampling performed at a single center study. Limitations of the culture portion include the lack of a broth enhancement step and lack of a 40^o^C plate to enhance recovery of *C. auris*. Future studies should be performed at multiple centers by multiple laboratories throughout the year to account for differences, such as the patient population and seasonal weather changes. Acknowledging these caveats, the current study showed that stick-mounted sponges have increased recovery of yeast compared to premoistened Eswabs. Furthermore, incubation at physiological as well as environmental temperatures increased the number of yeast isolates recovered.
